# Antimicrobial Resistance Profiling and Molecular Epidemiological Analysis of Extended Spectrum β-Lactamases Produced by Extraintestinal Invasive *Escherichia coli* Isolates From Ethiopia: The Presence of International High-Risk Clones ST131 and ST410 Revealed

**DOI:** 10.3389/fmicb.2021.706846

**Published:** 2021-08-02

**Authors:** Abebe Aseffa Negeri, Hassen Mamo, Jyoti M. Gurung, A. K. M. Firoj Mahmud, Maria Fällman, Eyasu Tigabu Seyoum, Adey Feleke Desta, Matthew S. Francis

**Affiliations:** ^1^National Clinical Bacteriology and Mycology Reference Laboratory, Ethiopian Public Health Institute, Addis Ababa, Ethiopia; ^2^Department of Microbial, Cellular and Molecular Biology, College of Natural and Computational Sciences, Addis Ababa University, Addis Ababa, Ethiopia; ^3^Department of Molecular Biology, Umeå University, Umeå, Sweden; ^4^Umeå Centre for Microbial Research (UCMR), Umeå University, Umeå, Sweden; ^5^Laboratory for Molecular Infection Medicine Sweden (MIMS), Umeå University, Umeå, Sweden

**Keywords:** Enterobacteriaceae, multidrug resistant, antibiotic susceptibility, multi-locus sequence typing, Bla CTX-M genes, community acquired infections

## Abstract

The treatment of invasive *Escherichia coli* infections is a challenge because of the emergence and rapid spread of multidrug resistant strains. Particular problems are those strains that produce extended spectrum β-lactamases (ESBL’s). Although the global characterization of these enzymes is advanced, knowledge of their molecular basis among clinical *E. coli* isolates in Ethiopia is extremely limited. This study intends to address this knowledge gap. The study combines antimicrobial resistance profiling and molecular epidemiology of ESBL genes among 204 *E. coli* clinical isolates collected from patient urine, blood, and pus at four geographically distinct health facilities in Ethiopia. All isolates exhibited multidrug resistance, with extensive resistance to ampicillin and first to fourth line generation cephalosporins and sulfamethoxazole-trimethoprim and ciprofloxacin. Extended spectrum β-lactamase genes were detected in 189 strains, and all but one were positive for CTX-Ms β-lactamases. Genes encoding for the group-1 CTX-Ms enzymes were most prolific, and CTX-M-15 was the most common ESBL identified. Group-9 CTX-Ms including CTX-M-14 and CTX-27 were detected only in 12 isolates and SHV ESBL types were identified in just 8 isolates. Bacterial typing revealed a high amount of strains associated with the B2 phylogenetic group. Crucially, the international high risk clones ST131 and ST410 were among the sequence types identified. This first time study revealed a high prevalence of CTX-M type ESBL’s circulating among *E. coli* clinical isolates in Ethiopia. Critically, they are associated with multidrug resistance phenotypes and high-risk clones first characterized in other parts of the world.

## Introduction

The emergence and spread of multidrug resistant strains of bacteria has made the treatment of *Escherichia coli* infections increasingly challenging across the world. This problem is exacerbated in sub-Saharan African counties, where robust laboratory diagnostics and surveillance systems for antimicrobial resistance are poorly developed despite the high incidence of extended spectrum β-lactamase (ESBL) producing bacteria (Williams et al., 2018). ESBL production refers to enzymes that confer the ability to hydrolyse the extended spectrum cephalosporins and monobactams, but not cephamycin such as cefoxitin and carbapenems. In Ethiopia specifically, the prevalence of ESBL-producing Enterobacteriaceae remains above 50% according to traditional phenotypic characterization (Beyene et al., 2019; Teklu et al., 2019). However, analyses of phenotypic data in the context of investigating the molecular mechanisms of ESBL-producing *E. coli* are limited.

However, in the global context, the established knowledge concerning the multidrug resistant strains that produce ESBLs is more expansive. For instance, genes for ESBL production are highly transmissible as a consequence of being encoded on plasmids and other mobile genetic elements and this rapidly expands the resistance spectrum when new antibiotics are introduced into clinical practice (Tooke et al., 2019). As these mobility elements often carry genes for resistance to other antimicrobial agents, ESBL-producing bacteria often present with multiple drug resistant (MDR) characteristics (Schwaber et al., 2005; Pitout, 2008). Additionally, ESBLs are classified into TEM and SHV ESBL variants and CTX-Ms. The TEM and SHV ESBL variants were the most common enzymes in the hospital setting during the 1980s and 1990s, with resistant *Klebsiella pneumoniae* particularly prolific (Castanheira et al., 2013; De Angelis et al., 2020). However, in recent decades CTX-Ms have emerged as the most common ESBL in both community and hospital environments throughout the world, with resistant *E. coli* as the most prolific (Paterson and Bonomo, 2005; Canton et al., 2012; Bevan et al., 2017; Peirano and Pitout, 2019; De Angelis et al., 2020).

CTX-M β-lactamase enzymes are classified into five groups based on their amino acid similarity: CTX-M group-1; CTX-M group-2; CTX-M group-8; CTX-M group-9; and CTX-M group-25. Within these five groups, CTX-M-15 (from group 1) and CTX-M-14 (group 9) are the most prevalent enzymes identified throughout the world (Canton et al., 2012; Bevan et al., 2017; De Angelis et al., 2020). The *bla*_CTM_ genes are often associated with genes conferring resistance to other antibiotic agents like fluoroquinolones and aminoglycosides (Bajaj et al., 2016). Examples of this are the pandemic clones ST131 and ST410, both widely distributed *E. coli* strains that efficiently spread in hospital and community settings (Nicolas-Chanoine et al., 2008; Woodford et al., 2011; Nicolas-Chanoine et al., 2014; Schaufler et al., 2016; Roer et al., 2018). The ST131 *E. coli* accounts for several extra-intestinal infections and asymptomatic humans can serve as a carrier (Nicolas-Chanoine et al., 2014; Forde et al., 2019) and like the ST131 strain; ST410 is responsible for various extra-intestinal infections and is resistant to fluoroquinolones and third-generation cephalosporins (Roer et al., 2018; Manges et al., 2019).

It is this understanding that we are trying to establish using isolates from Ethiopian patients, because, while the global characterization of these enzymes is advanced, knowledge of their molecular basis among clinical *E. coli* isolates in Ethiopia is extremely limited. Thus, this study investigated the molecular bases of resistance and the phylogenetic relationship of ESBL producing *E. coli* isolated from geographically distinct regions in Ethiopia. In doing so, we could also define the prevalence of international pandemic *E. coli* sequence type clones and their sub clones.

## Materials and Methods

### Bacterial Strain Collection and Initial Cataloguing

**FIGURE 1:**
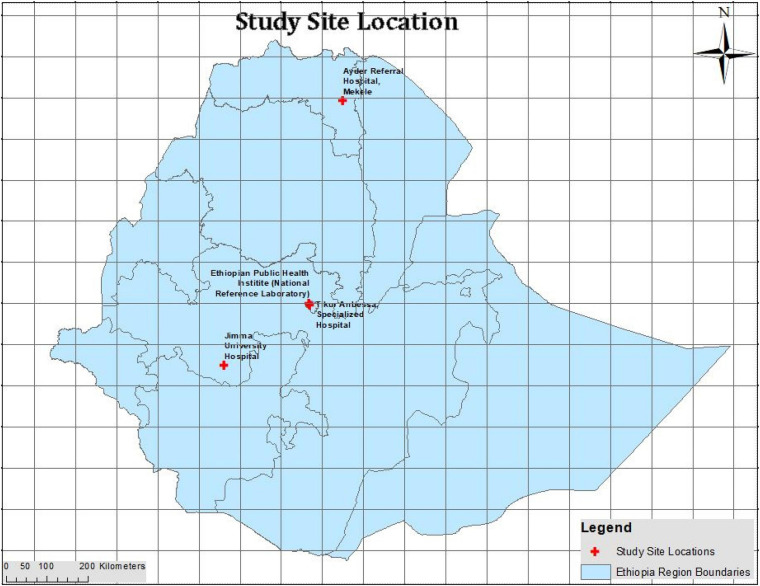
The four health facility sites engaged in this study and from where the isolates were obtained. The National reference laboratory and Tikur Abessa specialized hospital are located in the capital city, Addis Ababa. The Ayder referral hospital is situated in Mekele City in northern Ethiopia. The Jimma University Hospital lies in Jimma City, located in the south west of Ethiopia.

This study was conducted on 204 ESBL-producing *E. coli* isolates collected in 2018 from four health facilities in Ethiopia (Figure 1). These isolates were collected in line with the ongoing antimicrobial resistance surveillance which was launched in 2017 by the Ethiopian Public Health Institute under the supervision of the Federal Ministry of Health (Ibrahim et al., 2018). This national AMR surveillance is following the World Health Organization (WHO) Global Antimicrobial Resistance Surveillance System (GLASS) guideline and reports the phenotypic antimicrobial susceptibility profiles for priority AMR pathogens to the local, national and international stakeholders. All isolates were identified according to WHO guidelines operable in a basic clinical bacteriology laboratory for the identification of fermenter Gram-negative bacterial pathogens (Vandepitte et al., 2003). All isolates from the AMR sites are collected at the National Reference Laboratory (Addis Ababa) were they are stored at −80°C.

Critically, the national AMR surveillance is not tasked with characterizing the molecular mechanisms of antimicrobial resistance. Therefore, we used this substantial isolate collection as a resource to initiate a characterization of the molecular mechanisms behind ESBL production. For this study purpose, 204 non-duplicated *E. coli* strains resistant to at least one third generation cephalosporin were randomly selected and confirmed for ESBL production. The identity of these isolates was confirmed using a VITEK 2 compact automated ID/AST instrument according to the manufacturer’s instructions (bioMérieux, Marcy l’Etoile, France). All isolates were stored at the NRL at –80°C in trypticase soy broth containing 20% of glycerol.

### Antimicrobial Susceptibility Tests and Phenotypic Detection of ESBL Producing *E. coli*

The antimicrobial susceptibility tests and phenotypic detection of ESBL producing *E. coli* were based upon the standardized Kirby Bauer disk diffusion method on Mueller Hinton agar (Oxoid LTD, Basingstoke, and Hampshire, England) and the double disk synergy test, respectively. Each test was performed as recommended by the Clinical and Laboratory Standard Institute (CLSI) (Philadelphia, PA, United States) (Clinical and Laboratory Standards Institute, 2018). Bacterial isolates that were resistant to at least one agent in three different antimicrobial categories were recognised as MDR (Magiorakos et al., 2012). We used *E. coli* ATCC 25922 (ESBL negative) and *K. pneumoniae* subsp. pneumoniae ATCC 700603 (ESBL positive) as reference strains (Microbiologics Inc., Saint Cloud, MN, United States).

### Primers

Sigma-Aldrich Co (Dorset, England) synthesized all oligonucleotides.

### Molecular Characterization of β-Lactamases Producing *E. coli*

The entire strain collection was screened for the presence of bla_TEM,_ bla_SHV,_ bla_OXA_, and bla_CTX–M_ by multiplex PCR using primer combinations as previously described (Dallenne et al., 2010). Carbapenem resistant isolates were screened using multiplex PCR for the presence of bla_OXA48–like,_ bla_IMP_, bla_VIM_, and bla_KPC_ (Dallenne et al., 2010). All bla_SHV_ and bla_CTX–M_ and selected bla_TEM_ and bla_OXA_ positive PCR products were sequenced to determine the genetic variation within ESBL genes. The β-lactamase gene variants were identified by sequence alignment with known GenBank sequence using the Basic local alignment search tool (BLAST)^1^ (Altschul et al., 1990; Ye et al., 2006).

### Molecular Typing of Bacterial Strains

Phylogenetic grouping of ESBL producing *E. coli* was performed using Clermont’s revised PCR protocol (Clermont et al., 2013). All B2 phylogenetic group isolates were screened for international pandemic sequence type clones and their sub clones using established PCR protocols (Clermont et al., 2009; Johnson et al., 2009; Banerjee et al., 2013; Colpan et al., 2013).

A subset of 40 randomly selected strains, including 20 belonging to the ST131 PCR positive B2 group, were randomly selected and subjected to Multi-Locus Sequence Typing (MLST) according to the Achtman seven housekeeping genes (*adk*, *fumC*, *gyrB*, *icd*, *mdh*, *purA*, and *recA*) scheme as previously described (Wirth et al., 2006) and updated at https://enterobase.readthedocs.io/en/latest/mlst/mlst-legacy-info-ecoli.html. The 20 strains belonging to B2 phylogeny group with PCR positive indicator for ST131 were chosen to confirm the accuracy of the PCR procedure for specific detection of this high risk clone. The remaining 20 isolates belonging to different phylogeny groups were chosen as a pilot sample to gain a glimpse of the clonal distribution of *E. coli* in Ethiopia. When resources become available, this sample size will be increased to include all isolates.

Phylogenetic tree construction used Phyloviz software and the neighbour joining algorithm (Francisco et al., 2012). Tree branch-length minimization utilized the neighbour joining method of Saitou and Nei (Saitou and Nei, 1987). For the phylogenetic dendrogram, a WPGMA (Weighted Pair Group Method with Arithmetic mean) hierarchical clustering was performed by selecting the criterion of minimal dissimilarity. The default parameters of a 1% tolerance and an 85% similarity index were considered for clustering purposes.

### Data Analysis

The data was prepared using Excel spreadsheets (Microsoft Office) and imported to SPSS version 20.0. The frequencies of antimicrobial susceptibility and ESBL producers among different variables were calculated. Cross-tabulation was used to present the different relation between data. The compressions of ESBL distribution between studies sites and among clones were evaluated using the Chi-square test. The tests were two sided and *P* values < 0.05 were considered statistically significant.

### Ethical Considerations

Ethical clearance for this study was obtained from the Ethiopian Public Health Institute scientific and ethical review board (EPHI-IRB-054-2017) and Addis Ababa University, the College of Natural and Computational Science (IRB/039/2019).

## Results

### General Characteristics of the Bacterial Isolates

We characterized 204 *E. coli* ESBL producers from four health facilities in Ethiopia. In Table 1, we present the patient demographics associated with these isolates. A small majority of the isolates were recovered from female patients (*n* = 118, 57.8%), and from all age groups. The majority of isolates were recovered from urine (*n* = 166 at 81.4%) and pus (*n* = 30, 14.7%).

**TABLE 1:** Patient demographics associated with ESBL producing *E. coli* from Ethiopian health facilities.

**Variables**	**ESBL *E. coli* (%)^1^**
**Gender**	
Female	118 (57.8)
Male	86 (42.2)
**Age (years)**	
≤20	67 (32.8)
21–50	79 (38.7)
>50	58 (28.5)
**Specimen type**	
Urine	166 (81.4)
Blood	8 (3.9)
Pus	30 (14.7)
**Health facility sites^2^**	
NRL	72 (35.3)
TASH	69 (33.8)
ARH	30 (14.7)
JUH	33 (16.2)

*^1^n = 204 total number of isolates. In parentheses is the percent of isolates associated with each listed variable.*

*^2^NRL, National reference laboratory; TASH, Tikur Anbessa Specialized Hospital; ARH, Hyder Referral Hospiata; JUH, Jimma University hospital (see Figure 1 for physical geographical location).*

### Measured Antimicrobial Susceptibility Profiles

**FIGURE 2:**
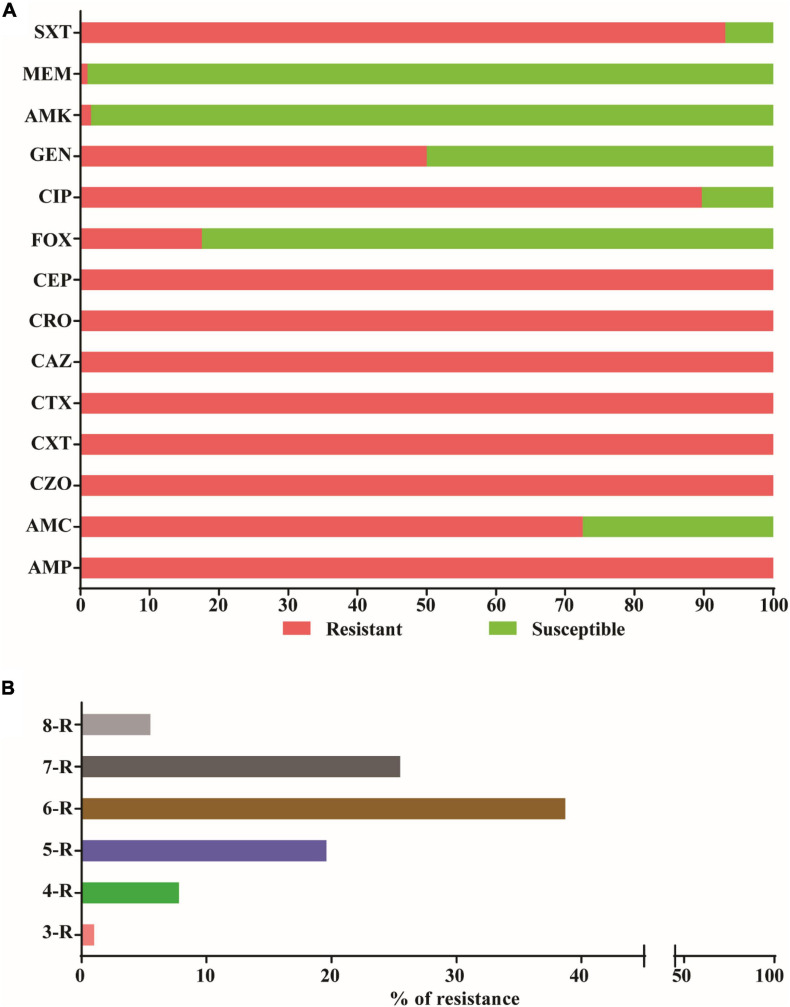
Antibiotic susceptibility profile and multidrug resistance patterns. **(A)** The percent resistance of 204 ESBL positive *E. coli* isolates from four health facilities in Ethiopia according to the CLSI disk diffusion breakpoints. Resistance was defined as isolates with intermediate resistance and complete resistance inhibition zone size. Antibiotics tested were ampicillin (AMP), amoxicillin-clavulanate (AMC), cefazolin (CZO), cefuroxime (CXM), cefotaxime (CTX), ceftazidime (CAZ), Ceftriaxone (CRO), cefepime (CEF), cefoxitine (FOX), ciprofloxacin (CIP), Amikacin (AMK), gentamicin (GEN), Meropenem (MER), and Sulphamethoxazole-Trimethoprim (SXT). **(B)** The percent resistance to three or more categories of antibiotics among the 204 ESBL producing *E. coli*. The numbers 3–8 represent the number antimicrobial categories and R stands for resistance.

Antimicrobial susceptibility testing revealed that all 204 ESBL producers in the strain collection were resistant to ampicillin and all generations (first to fourth) of cephalosporins, but remained highly susceptible to meropenem (*n* = 202, 99%) and amikacin (*n* = 201, 98.5%) (Figure 2A). Moreover, 93.1% (*n* = 190) of *E. coli* strains were resistant to sulfamethoxazole-trimethoprim and 89.7% (*n* = 183) to ciprofloxacin (Figure 2A). In addition, the prevalence of resistance to amoxicillin-clavulanate, gentamicin and cefoxitin were 72.5% (*n* = 148), 50% (*n* = 102), and 17.6% (*n* = 36), respectively (Figure 2A). Significantly, all 204 isolates were resistant to more than one agent in three separate antimicrobial categories (Figure 2B), which is indicative of MDR according to Magiorakos and colleagues (Magiorakos et al., 2012). In fact, most isolates were resistant to antibiotics belonging to six or seven antimicrobial categories (Figure 2B).

### Molecular Characterization β-Lactamase Genes

All 204 *E. coli* strains with ESBL phenotype were characterised genetically to identify the β-lactamase gene type. We identified at least one β-lactamase gene in all 204 isolates. CTX-M group-1 was detected in 94.7% (*n* = 179) of all CTX-M positive isolates (*n* = 188) (Table 2). Of these, 87.7% (*n* = 157) harboured the CTX-M-15 subtype (Table 2). The non-ESBL TEM-1 and OXA-1 β-lactamase genes were detected in these CTX-M-15 positive isolates at a frequency of 72.2% (*n* = 115) and 82.2% (*n* = 129), respectively (Table 2). Of the remaining isolates with a detectable group-1 CTX-M (*n* = 22, 12.3%), we could specify CTX-M-55, CTX-M-101, CTX-M-103, CTX-M-142, CTX-M-180, CTX-M-182, CTX-M-225, and CTX-M-227 types (Table 2). One isolate was even positive for both CTX-M-182 and CTX-M-227. In addition, CTX-M group-9 was detected in 6.4% (*n* = 12) of the 188 CTX-M positive strains, of which 91.7% (*n* = 11) harboured CTX-M-27, while the remaining isolate was positive for CTX-M-14 (Table 2). Two CTX-M-27 isolates were also positive for CTX-M-15. Our analysis also revealed eight SHV positive isolates, of which 62.5% (*n* = 5) contained SHV-11 and the remaining three isolates contained one of SHV-7, SHV-34 or SHV-61, respectively (Table 2). Association with CTX-M-15 was found in these isolates excepting the SHV-61 isolate. Finally, the *bla*_OXA–1_ and *bla*_TEM–1_ genes were identified at a respective frequency of 78.9% (*n* = 161) and 73.5% (*n* = 150) (Table 2). Conversely, no carbapenemase genes were detected despite meropenem resistance in two isolates. However, one of these isolates harboured genes for both CTX-M-15 and CTX-M-27, and the other genes for both CTX-M-182 and CTX-M-27. According to others (Tsai et al., 2013; Wang et al., 2016), being positive for one CTX-M from group-1 and CTX-M-27 from group-9 might be a reason these two isolates are resistant to carbapenem. However, resistance might also be due to porin loss in combination with ESBLs or AmpC β-lactamase production (Doumith et al., 2009).

**TABLE 2:** Type and distribution of β-lactamases detected among ESBL-producing *E. coli* isolates from the four health facilities.

**β-Lactamases**	**Number of isolates (%)^1^**					
	**Total**	**NRL**	**TASH**	**ARH**	**JUH**	***P*-value**
CTX- M	188	69	65	28	26	
Group-1 CTX-M	179	64	62	27	26	
CTX-M-15	157 (87.7)	53 (82.8)	55 (88.7)	23 (82.1)	26 (100)	0.255
CTX-M-55	2 (1.1)	1 (1.6)	0 (0.0)	1 (3.7)	0 (0.0)	
CTX-M-101	3 (1.7)	3 (4.7)	0 (0.0)	0 (0.0)	0 (0.0)	
CTX-M-103	1 (0.6)	0 (0.0)	1 (1.6)	0 (0.0)	0 (0.0)	
CTX-M-142	1 (0.6)	0 (0.0)	1 (1.6)	0 (0.0)	0 (0.0)	
CTX-M-180	5 (2.8)	4 (6.3)	0 (0.0)	1 (3.7)	0 (0.0)	
CTX-M-182	6 (3.4)	2 (3.1)	3 (4.8)	1 (3.7)	0 (0.0)	
CTX-M-225	3 (1.7)	1 (1.6)	2 (3.3)	0 (0.0)	0 (0.0)	
CTX-M-227	1 (0.6)	0 (0.0)	0 (0.0)	1 (3.7)	0 (0.0)	
Group-9 CTX-M	12	5	5	1	1	
CTX-M-14	1 (8.3)	1 (20.0)	0 (0.0)	0 (0.0)	0 (0.0)	
CTX-M-27	11 (91.7)	4 (80.8)	5 (100)	1 (100)	1 (100)	
SHV	8	0	2	1	5	
SHV-7	1 (12.5)	0 (0.0)	1 (50.0)	0 (0.0)	0 (0.0)	
SHV-11	5 (62.5)	0 (0.0)	1 (50.0)	0 (0.0)	4 (80.0)	
SHV-34	1 (12.5)	0 (0.0)	0 (0.0)	1 (100)	0 (0.0)	
SHV-61	1 (12.5)	0 (0.0)	0 (0.0)	0 (0.0)	1 (20.0)	
TEM-1	150	52	53	19	26	
OXA-1	161	57	54	23	27	

*^1^The percentage was calculated for each health facility site and the *P*-value determined to evaluate the distribution of CTX-M-15 per site.*

Crucially, β-lactamases were detected in isolates obtained from all four study sites. Focusing on the frequently identified CTX-M-15 type, we observed no significant difference (*P* > 0.05) in the distribution frequency of this type among the four study sites (Table 2). However, we note that 81.8% (*n* = 9) of CTX-M-27 positive isolates originated from the geographically linked NRL and TASH, while 65.5% (*n* = 5) of SHV positive isolates originated from JUH (Table 2). Whether these associations are significant is unclear given the low numbers of isolates in these categories.

### Phylogenetic Grouping of β-Lactamase-Producing *E. coli*

Next we applied the modified Clermont quadruplex PCR protocol (Clermont et al., 2013) to assign 97.0% (*n* = 198) of the 204 isolates into known phylogenetic groups. The largest phylogenetic group was B2 occurring at a frequency of 44.9% (*n* = 89) (Table 3). Other phylo-groups were detected at the frequencies of 24.7% for group A (*n* = 49), 16.7% for group C (*n* = 33), 4.5% for group D (*n* = 9), 3.5% for group F (*n* = 7) and 3.0% for group B1 (*n* = 6) (Table 3). The production of group 1 CTX-Ms, including CTX-M-15, were detected in all phylogenetic groups, with the highest detection rate in phylo-group B2 (43.9%, *n* = 69) (Table 3). On the other hand, group-9 CTX-M distribution was restricted to phylo-groups A, B2 and C, although phylo-group B2 dominated (66.7%, *n* = 8) (Table 3). Phylo-group B2 was also most commonly associated with isolates containing bla_OXA–1_ (47.2%, *n* = 76) and bla_TEM–1_ (42.7%, *n* = 64) (Table 3).

**TABLE 3:** The phylogenetic distribution of ESBL producing *E. coli* isolated from clinical samples originating four health facilities in Ethiopia.

**Phylogenetic group**	**Number of isolates (%) per β-lactamase gene type**					
	**CTX-M-15 (*n* = 157)**	**Other Group-1 CTX-Ms (*n* = 22)**	**Group-9 CTX-Ms (*n* = 12)**	**SHV (*n* = 8)**	**TEM-1 (*n* = 150)**	**OXA-1 (*n* = 161)**
A^b^	41 (26.1)	3 (13.6)	3 (25.0)	2 (25.0)	34 (22.7)	38 (23.6)
B1	4 (2.6)	2 (9.1)	0	0	8 (5.3)	4 (2.5)
B2^a,b^	69 (43.9)	8 (36.4)	8 (66.7)	4 (50.0)	64 (42.7)	76 (47.2)
C^a,b^	28 (17.8)	3 (13.6)	1 (8.3)	1 (12.5)	26 (17.3)	25 (15.5)
D	6 (3.8)	3 (13.6)	0	0	9 (6.0)	8 (5.0)
F^b^	4 (2.6)	2 (9.2)	0	1 (12.5)	5 (3.3)	6 (3.7)
U	5 (3.2)	1 (4.4)	0	0	4 (2.7)	4 (2.5)

*^a^Two isolates belonging to phylogenetic B2 and one isolate in phylogenetic group C were detected for two variants of CTX-Ms.*

*^b^Two group A, three group B2, one group C, and 1 group F were detected for CTX-M-15 and variants of SHV.*

### Prevalence of *E. coli* Sequence Type (ST) 131 Clonal Group and H30 and H30-Rx Subclones

We further characterized the B2 phylo-group to provide novel information on the occurrence and distribution of the international pandemic ST131 clonal group and its sub-clones. Out of 89 isolates within phylo-group B2, 83.1% (*n* = 74) were positive for the *mdh* and *gyrB* ST131 markers. Of these 74 isolates, serotype O25b accounted for 83.8% (*n* = 62), and all of these 62 were positive for *fimH*30. Of these, 67.7% (*n* = 42) were identified as the H30Rx clade.

**FIGURE 3:**
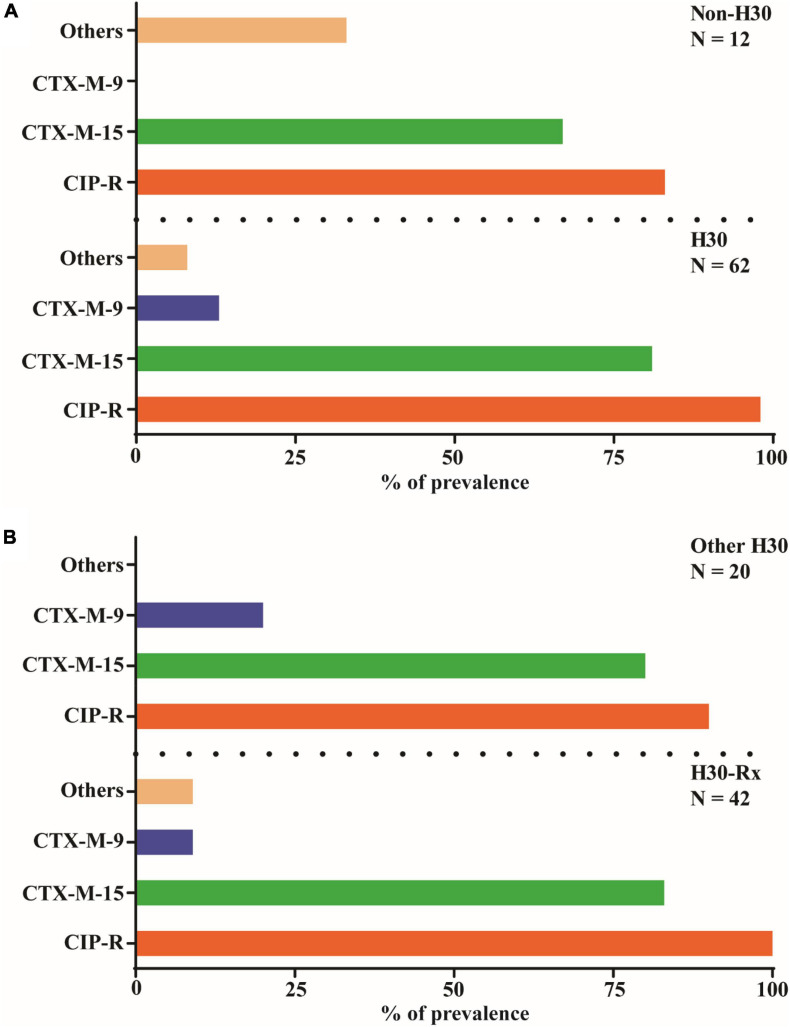
Prevalence of key CTX-M genetic variants and ciprofloxacin resistance among 74 ST131 isolates. Note that as with all strains in this study the ST131 isolates were confirmed producers of β-lactamases as measured by resistance ampicillin (see Figure 2A). **(A)** All 74 ST131 isolates classified into H30 subclone (*n* = 62) and non-H30 subclone (*n* = 12). These 74 isolates were then tested for the prevalence of CTX-M gene distribution following PCR amplification and sequencing. Most of the isolates harboured either the gene encoding for the β-lactamase enzyme CTX-M-15 or CTX-M-9. A Pearson Chi-square test was used to evaluate if this distribution is statistically significant. The CTX-Ms distribution profile among the isolates of the two different groups were not significantly different (*P* > 0.05). **(B)** Of the 62 H30 isolates, 67.7% (*n* = 42) were identified as H30Rx using PCR. The vast majority of all isolates were ciprofloxacin resistant (CIP-R), CTX-Ms in group 1 other than CTX-M-9 or CTX-M-15 are classified collectively as “other.”

To examine for the prevalence of genes encoding the different CTX-M β-lactamases as well as the extent of ciprofloxacin (fluoroquinolone) resistance among the ST131 isolates (*n* = 74), we first classified them into ST131-H30 (*n* = 62) and non-ST131-H30 (*n* = 12) (Figure 3A). Interestingly, the PCR amplification and sequencing of the isolates revealed a high prevalence of the CTX-M-15 encoding gene, and to a lesser extent the CTX-M-9 encoding gene. There was no significant difference (*P* > 0.05) in prevalence of group-1 CTX-Ms, including CTX-M-15, between these two groups (Figure 3A). On the other hand, group-9 CTX-Ms genes, including the gene encoding CTX-M-9, were all associated with ST131-H30 isolates (Figure 3A). Although both bacterial groups were resistant to ciprofloxacin, the ST131-H30 group were significantly more resistant (*P* < 0.05) than the non-ST131-H30 group (Figure 3A). We also classified the most prevalent ST131-H30 (*n* = 62) subclone into H30-Rx (*n* = 42) and other H30 (*n* = 20), and observed that genes encoding group-1 CTX-M’s, including CTX-M-15, were detected in both groups with similar frequency (Figure 3B). However, group-9 CTX-M’s were more commonly associated with other H30 strains compared to H30-Rx (*P* < 0.05) (Figure 3B). Moreover, the patterns of ciprofloxacin (fluoroquinolone) resistance were more pronounced in H30-Rx strains (*P* < 0.05) (Figure 3B).

### Multi-Locus Sequence Typing

**FIGURE 4:**
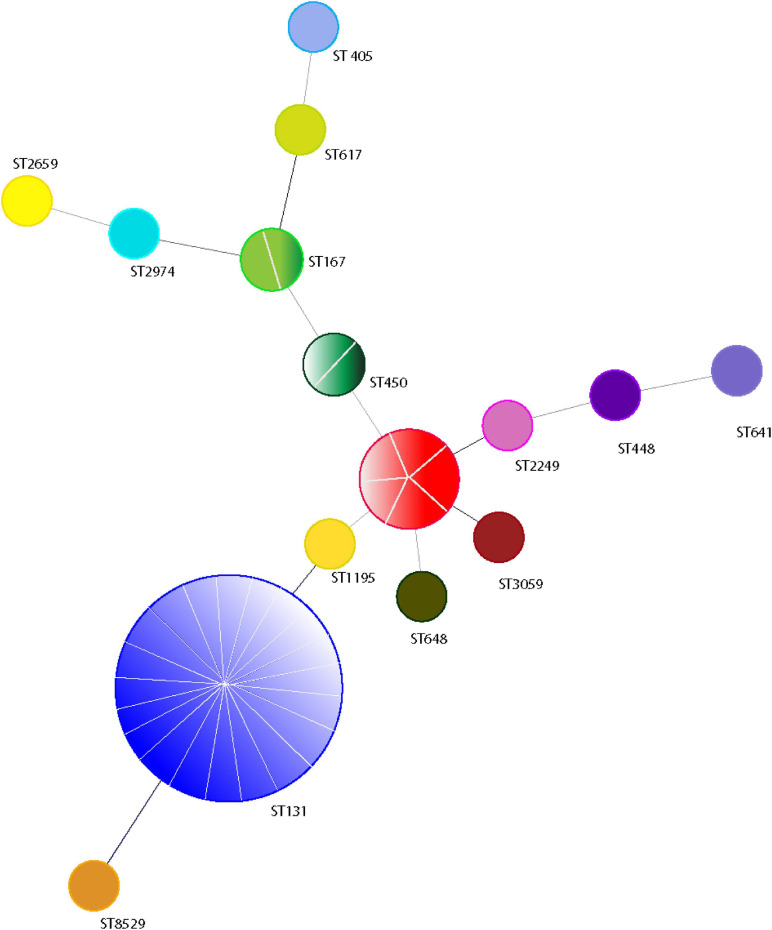
eBURST analysis of minimum spanning tree of 40 ESBL positive *E. coli* isolates based on MLST genotypes. The genotypes represented by a circle and the line between each circle represents individual isolates. The line within a circle indicates the number of isolates belong to the genotype.

**FIGURE 5:**
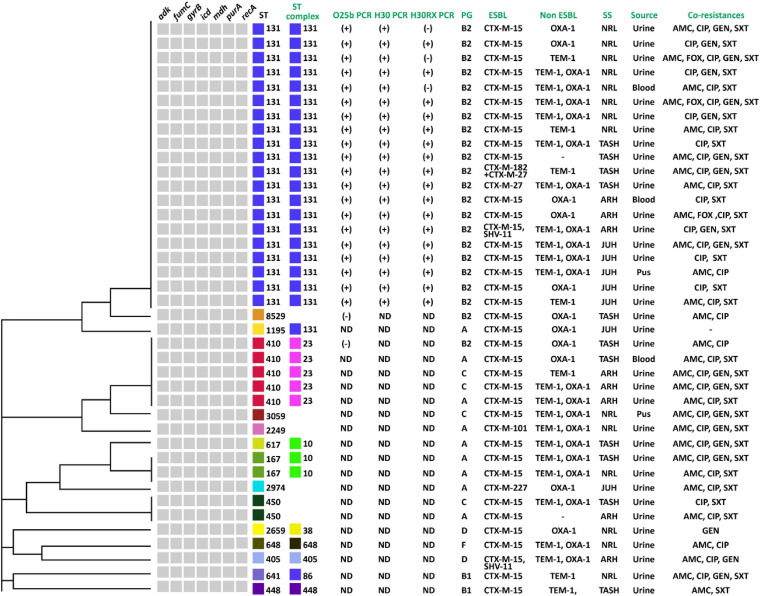
UPGMA dendrogram showing the genetic relationships of STs of 40 ESBL positive *E. coli* strains from MLST data along with different variables: the ST131 PCR results for O25b, H30, and H30Rx; the phylogenetic groups (PG); type of ESBLs and non-ESBLs; study sites (SS); clinical specimens (sources) and resistance to non-cephalosporin antibiotics and ampicillin (co-resistance). ND, not done, AMC, amoxicillin-clavulanate, FOX, cefoxitine, CIP, ciprofloxacin, GEN, gentamicin, and SXT, Sulphamethoxazole-Trimethoprim.

**FIGURE 6:**
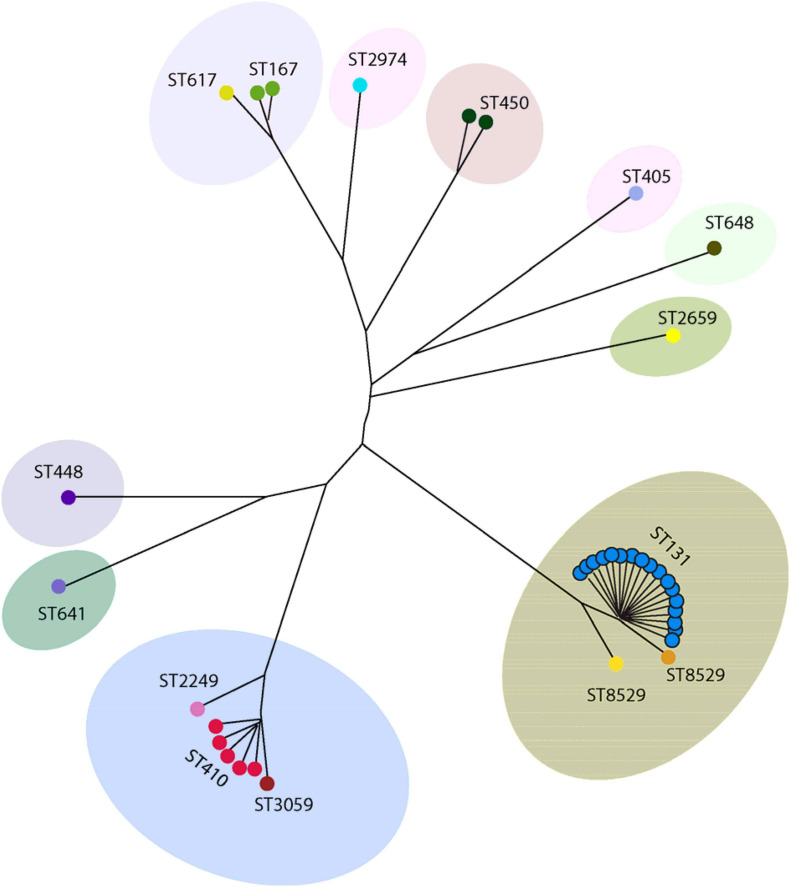
Phylogenetic distribution of 40 ESBL producing *E. coli* according their sequence types. The phylogenetic construction was done using Phyloviz software based on the seven MLST gene sequences. The sequence type with single locus variants are grouped together and indicated in a single circle.

Forty isolates belonging to different phylogenetic groups were selected for MLST analysis. We detected 15 different sequence types (ST) using the allelic profile of seven housekeeping genes (*adk*, *fumC*, *gyrB*, *icd mdh*, *purA*, and *recA*). The most dominant ST identified was ST131 (50.0%, *n* = 20) followed by ST410 (12.5%, *n* = 5) (Figure 4). ST167 and ST450 were detected in two isolates, while a further eleven STs were identified for the remaining isolates (Figure 4). A BLAST analysis using the Enterobase database (Zhou et al., 2020) classified the 15 ST’s into eight sequence complexes (SCs) or clonal complexes (CCs) in which 21 (52.5%) belonged to CC131 (SC131), 5 (12.5%) to CC23, and 3 (7.5%) to CC10 (Figure 5). Five isolates were identified as CC38, CC86, CC405, CC440, and CC648 (Figure 5). On the other hand, the allelic profile of ST8529, ST3059, ST2259, ST2974, and ST450 were branded as singletons, as these STs could not be assigned to any SC or CC within the Enterobase database (Figure 5). Based on these CC assignments, ST networks were constructed using the PHYLOVIZ software^2^ (Francisco et al., 2012). The epidemiological links and phylogenetic relationships among the STs are illustrated in Figure 6.

CC131 was most prevalent and contained all ST131 strains. These were positive for O25b and H30 subclone specific PCR, and 85% (*n* = 17) were positive for H30-Rx subclone specific PCR (Figure 5). All belonged to phylo-group B2, and 90% (*n* = 18) were positive for CTX-M-15, 5% (*n* = 1) positive for CTX-M-27, and 5% (*n* = 1) positive for both CTX-M-182 and CTX-M-27 (Figure 5). One isolate was also positive for SHV-11 (Figure 5). Most ST131 isolates were recovered from urine samples, but exhibited variable resistance levels to the non-cephalosporin antibiotics, as well as non-ESBL enzyme OXA-1 and TEM production (Figure 5). CC23 was also prevalent, and to which all ST410 strains belonged. All produced CTX-M-15; despite belonging to different phylogenetic groups, but not all produced non-ESBL enzymes, and they displayed variable resistance levels to non-cephalosporine antibiotics (Figure 5). All except one isolate was recovered from urine. Finally, CC10 was also prevalent, and comprised one ST617 and two ST167 isolates. All were in phylo-group A, and all were positive for CTX-M-15 and the non-ESBL enzymes OXA-1 and TEM (Figure 5).

## Discussion

This study represents the first phenotypic and genotypic correlation study of multidrug resistant, ESBL producing *E. coli* obtained from geographically distinct areas in Ethiopia. It includes characterization of the phylogenetic distribution and the role of *E. coli* clonal distribution in antimicrobial resistance in Ethiopia. The prevalence of multidrug resistance among Gram-negative bacteria has been previously reported for Ethiopia (Beyene et al., 2019), as well as in other African nations (Ramadan et al., 2019; Hassuna et al., 2020). However, herein all 204 isolates were multidrug resistant. This substantial number is an obvious point of difference between Ethiopia and developed countries. Hence, this study lays the foundation for addressing many important questions concerning the structure of *E. coli* populations in Ethiopia, on their pathogenic potential, and the factors responsible for the prevalence of multidrug resistance.

In accordance with global reports, *bla*_CTX–M_ was the most prominent β-lactamase gene identified in our isolate collection. Of these, *bla*_CTX–M–15_ was the most abundant variant, corroborating an earlier finding from a study based on a single hospital in Ethiopia (Zeynudin et al., 2018). Although CTX-M-15 is already a globally distributed enzyme (Smet et al., 2010; Chandramohan and Revell, 2012; Denisuik et al., 2013; Brolund et al., 2014; Wang et al., 2016; Pietsch et al., 2017; Ramadan et al., 2019; Quinones et al., 2020), our findings indicate that the burden of this enzyme is disproportionately higher in Ethiopia. Hence, there is a pressing need to focus international attention on the African continent in order to tackle the global spread of AMR pathogens. While reasons for the rapid emergence of dominant ESBL genotypes are well documented for developed nations (Wellington et al., 2013; Pehrsson et al., 2016; Bevan et al., 2017). This has not been investigated in Ethiopia. Therefore, a comprehensive molecular characterization of antimicrobial resistant genes from bacterial strains derived from hospital, community, and agricultural sources is needed. This can only be achieved with international cooperation. Indeed, political leaders and major international health organizations universally advocate the philosophy of international cooperation under the mantra that no-one is safe until everyone is safe.

Phylo-grouping of *E coli* provides essential evidence concerning a strain’s phenotypic and genotypic features, ecological niche, life history, and pathogenic potential (Tenaillon et al., 2010). For example, phylo-group B2 and D are prominent extra-intestinal human pathogenic *E. coli* (Kazemnia et al., 2014; Tong et al., 2014) and cause more significant infection than any other phylogenetic group (Lee et al., 2016). Moreover, phylo-group A are commonly commensal and/or intestinal pathogens (Clermont et al., 2011), but are also occasionally associated with extra-intestinal urinary tract infections (Campos et al., 2018). We assigned 97% of the ESBL producing *E. coli* in our Ethiopian strain collection to six known phylogenetic groups. Consistent with the clinical origin of our strain collection, phylo-group B2 dominated, followed by phylo-group A. This suggests that many of these isolates have significant pathogenic potential; this will be investigated in the future.

It is worth noting that we identified a prevalence of strains belonging to phylo-group C, which is not typically associated with harmful infections (Lee et al., 2016). This might indicate that our strain collection contains a number of isolates with an animal origin, and have undergone a regional cross transmission from animals to humans. We have not tested this here, but the idea is supported by studies that have demonstrated high cross transmission risks in regions afflicted by poor sanitation infrastructure (Wellington et al., 2013; Pehrsson et al., 2016). Moreover, phylo-group C strains derived from humans and animals can share common genetic features (Clermont et al., 2011), a trait that is over-represented in developing countries like Ethiopia (Asante et al., 2019).

This study did not reveal any obvious correlation between ESBL production and phylogenetic groups. In fact, ESBL genes were widely disseminated across all phylogenetic groups. An obvious explanation for this is the excessive antibiotic exposure in the gastro-intestinal tract, which selects for antibiotic resistance gene carriage by extra-intestinal pathogenic *E. coli*, as well as for the efficient horizontal transfer of plasmids containing ESBL genes among *E. coli* populations. Moreover, we identified for the first time in Ethiopia a high prevalence of the international high risk pandemic clones ST131 and ST410. Pandemic clones are a dominant source for the maintenance and propagation of genes responsible for antimicrobial resistance and therefore play an important role in dissemination of ESBL genes in *E. coli* populations, especially within the B2 phylogenetic group (Peirano et al., 2014).

In fact, our MLST analysis demonstrated 15 phylogenetic related ST’s among 40 randomly selected isolates, but the ST131 type dominated our findings. This over representation is likely caused by its widespread geographical distribution (Nicolas-Chanoine et al., 2008; Woodford et al., 2011; Nicolas-Chanoine et al., 2014). ST131 strains are usually subtyped as 025b (Matsumura et al., 2012; Banerjee et al., 2013; Dahbi et al., 2013; Nicolas-Chanoine et al., 2014; Hojabri et al., 2017; Chen et al., 2019). A high prevalence of 025b subtype ST131 strains in our phylo-group B2 is entirely consistent with this. All of the identified O25b-ST131 isolates were the H30 subclone, of which H30-Rx represented a major subset. This is critical because this subset are extensively resistant to fluoroquinolone (Johnson et al., 2017). Furthermore, there were no differences in CTX-M-15 production between H30 and non-H30 ST131 isolates. This contrasted previous studies where differences in CTX-M production in these two groups were reported (Banerjee et al., 2013; Chen et al., 2019). All our isolates were ESBL producers, which may explain the similar CTX-M production profiles among our ST131 population. The high proportion of CTX-M-27 production among our ST131 isolates is also worth noting since it upholds a new trend emerging around the world (Birgy et al., 2019).

The second most prevalent sequence type, ST410, is an *E. coli* clone currently recognized as an international high risk clone on par with ST131 (Schaufler et al., 2016). This type has high transmissibility between human, animals, and the environment (Falgenhauer et al., 2016; Schaufler et al., 2016), with a capacity to acquire genes encoding resistance to diverse antimicrobial categories (Liu et al., 2015; Falgenhauer et al., 2016; Piazza et al., 2018; Roer et al., 2018). The third most prevalent *E. coli* clone we identified belonged to the CC10 complex. The CC10 complex is a common faecal commensal of both animal and humans, can cause human ExPEC infections, inhabit diverse environmental niches and carry a wide variety of resistance-associated plasmids (Reid et al., 2019). Hence, the epidemiology of this lineage should be also closely monitored in Ethiopia.

In summary, to our knowledge this represents the first report of these clones in Ethiopia. Critically, ST131 and ST410 high-risk clones are circulating in Ethiopia with high frequency. This has major implications to Ethiopian public health. To help address this, follow-up work must focus on molecular epidemiological analysis to investigate their source, local burden and distribution. Moreover, molecular epidemiological analysis of larger isolate collections is required to appreciate all ST circulating in this region.

## Data Availability Statement

The raw data supporting the conclusions of this article will be made available by the authors, without undue reservation.

## Ethics Statement

The studies involving human participants were reviewed and approved by Ethiopian Public Health Institute Scientific and Ethical Review Board (EPHI-IRB-054-2017), Addis Ababa University, the College of Natural and Computational Science (IRB/039/2019). Written informed consent to participate in this study was provided by the participants’ legal guardian/next of kin.

## Author Contributions

AN, HM, AD, and MFr conceived and designed the study and wrote the manuscript. AN, JG, ES, and AM performed the experiments. MF and MFr provided essential resources. AN, ES, and MFr analysed the data. All authors contributed to the manuscript revision and approved the final manuscript.

## Conflict of Interest

The authors declare that the research was conducted in the absence of any commercial or financial relationships that could be construed as a potential conflict of interest.

## Publisher’s Note

All claims expressed in this article are solely those of the authors and do not necessarily represent those of their affiliated organizations, or those of the publisher, the editors and the reviewers. Any product that may be evaluated in this article, or claim that may be made by its manufacturer, is not guaranteed or endorsed by the publisher.

## Footnotes

1
http://www.ncbi.nlm.nih.gov/BLAST/

2
http://www.phyloviz.net/

## References

[B1] Altschul S. F. Gish W. Miller W. Myers E. W. Lipman D. J. (1990). Basic local alignment search tool. *J. Mol. Biol.* 215 403–410. 2231712 10.1016/S0022-2836(05)80360-2

[B2] Asante J. Noreddin A. El Zowalaty M. E. (2019). Systematic review of important bacterial zoonoses in africa in the last decade in light of the ‘one health’ concept. *Pathogens* 8:50. 10.3390/pathogens8020050 30995815 PMC6631375

[B3] Bajaj P. Singh N. S. Virdi J. S. (2016). *Escherichia coli* beta-lactamases: what really matters. *Front. Microbiol.* 7:417. 10.3389/fmicb.2016.00417 27065978 PMC4811930

[B4] Banerjee R. Robicsek A. Kuskowski M. A. Porter S. Johnston B. D. Sokurenko E. (2013). Molecular epidemiology of *Escherichia coli* sequence type 131 and Its H30 and H30-Rx subclones among extended-spectrum-beta-lactamase-positive and -negative E. coli clinical isolates from the Chicago Region, 2007 to 2010. *Antimicrob. Agents Chemother.* 57 6385–6388. 10.1128/aac.01604-13 24080662 PMC3837873

[B5] Bevan E. R. Jones A. M. Hawkey P. M. (2017). Global epidemiology of CTX-M beta-lactamases: temporal and geographical shifts in genotype. *J. Antimicrob. Chemother.* 72 2145–2155. 10.1093/jac/dkx146 28541467

[B6] Beyene D. Bitew A. Fantew S. Mihret A. Evans M. (2019). Multidrug-resistant profile and prevalence of extended spectrum beta-lactamase and carbapenemase production in fermentative gram-negative bacilli recovered from patients and specimens referred to national reference laboratory, Addis Ababa, Ethiopia. *PLoS One* 14:e0222911. 10.1371/journal.pone.0222911 31553773 PMC6760794

[B7] Birgy A. Levy C. Nicolas-Chanoine M. H. Cointe A. Hobson C. A. Magnan M. (2019). Independent host factors and bacterial genetic determinants of the emergence and dominance of *Escherichia coli* sequence type 131 CTX-M-27 in a community pediatric cohort study. *Antimicrob. Agents Chemother.* 63:e00382. 31085515 10.1128/AAC.00382-19 PMC6591637

[B8] Brolund A. Edquist P. J. Makitalo B. Olsson-Liljequist B. Soderblom T. Wisell K. T. (2014). Epidemiology of extended-spectrum beta-lactamase-producing *Escherichia coli* in Sweden 2007-2011. *Clin. Microbiol. Infect.* 20 O344–O352. 24118431 10.1111/1469-0691.12413

[B9] Campos A. C. C. Andrade N. L. Ferdous M. Chlebowicz M. A. Santos C. C. Correal J. C. D. (2018). Comprehensive molecular characterization of *Escherichia coli* Isolates from urine samples of hospitalized patients in rio de Janeiro, Brazil. *Front. Microbiol.* 9:243. 10.3389/fmicb.2018.00243 29503639 PMC5821075

[B10] Canton R. Gonzalez-Alba J. M. Galan J. C. (2012). CTX-M enzymes: origin and diffusion. *Front. Microbiol.* 3:110. 10.3389/fmicb.2012.00110 22485109 PMC3316993

[B11] Castanheira M. Farrell S. E. Deshpande L. M. Mendes R. E. Jones R. N. (2013). Prevalence of beta-lactamase-encoding genes among *Enterobacteriaceae* bacteremia isolates collected in 26 U.S. hospitals: report from the SENTRY antimicrobial surveillance program (2010). *Antimicrob. Agents Chemother.* 57 3012–3020. 10.1128/aac.02252-12 23587957 PMC3697373

[B12] Chandramohan L. Revell P. A. (2012). Prevalence and molecular characterization of extended-spectrum-beta-lactamase-producing *Enterobacteriaceae* in a pediatric patient population. *Antimicrob. Agents Chemother.* 56 4765–4770. 10.1128/aac.00666-12 22733062 PMC3421901

[B13] Chen S. L. Ding Y. Apisarnthanarak A. Kalimuddin S. Archuleta S. Omar S. F. S. (2019). The higher prevalence of extended spectrum beta-lactamases among *Escherichia coli* ST131 in Southeast Asia is driven by expansion of a single, locally prevalent subclone. *Sci. Rep.* 9: 13245. 31519972 10.1038/s41598-019-49467-5 PMC6744567

[B14] Clermont O. Christenson J. K. Denamur E. Gordon D. M. (2013). The clermont *Escherichia coli* phylo-typing method revisited: improvement of specificity and detection of new phylo-groups. *Environ. Microbiol. Rep.* 5 58–65. 10.1111/1758-2229.12019 23757131

[B15] Clermont O. Dhanji H. Upton M. Gibreel T. Fox A. Boyd D. (2009). Rapid detection of the O25b-ST131 clone of *Escherichia coli* encompassing the CTX-M-15-producing strains. *J. Antimicrob. Chemother.* 64 274–277. 10.1093/jac/dkp194 19474064

[B16] Clermont O. Olier M. Hoede C. Diancourt L. Brisse S. Keroudean M. (2011). Animal and human pathogenic *Escherichia coli* strains share common genetic backgrounds. *Infect. Genet. Evol.* 11 654–662. 10.1016/j.meegid.2011.02.005 21324381

[B17] Clinical and Laboratory Standards Institute (2018). *M100. Performance Standards for Antimicrobial Suceptibility Testing.* Wayne, IL: Clinical and Laboratory Standards Institute.

[B18] Colpan A. Johnston B. Porter S. Clabots C. Anway R. Thao L. (2013). *Escherichia coli* sequence type 131 (ST131) subclone H30 as an emergent multidrug-resistant pathogen among US veterans. *Clin. Infect. Dis.* 57 1256–1265. 10.1093/cid/cit503 23926176 PMC3792724

[B19] Dahbi G. Mora A. Lopez C. Alonso M. P. Mamani R. Marzoa J. (2013). Emergence of new variants of ST131 clonal group among extraintestinal pathogenic *Escherichia coli* producing extended-spectrum beta-lactamases. *Int. J. Antimicrob. Agents* 42 347–351. 10.1016/j.ijantimicag.2013.06.017 23992646

[B20] Dallenne C. Da Costa A. Decre D. Favier C. Arlet G. (2010). Development of a set of multiplex PCR assays for the detection of genes encoding important beta-lactamases in *Enterobacteriaceae*. *J. Antimicrob. Chemother.* 65 490–495. 10.1093/jac/dkp498 20071363

[B21] De Angelis G. Del Giacomo P. Posteraro B. Sanguinetti M. Tumbarello M. (2020). Molecular mechanisms, epidemiology, and clinical importance of beta-lactam resistance in *Enterobacteriaceae*. *Int. J. Mol. Sci.* 21:5090. 10.3390/ijms21145090 32708513 PMC7404273

[B22] Denisuik A. J. Lagace-Wiens P. R. Pitout J. D. Mulvey M. R. Simner P. J. Tailor F. (2013). Molecular epidemiology of extended-spectrum beta- lactamase-, AMPC beta-lactamase- and carbapenemase-producing *Escherichia coli* and *Klebsiella pneumoniae* isolated from canadian hospitals over a 5 year period: CANWARD 2007-11. *J. Antimicrob. Chemother.* 68 Suppl 1 i57–i65. 23587779 10.1093/jac/dkt027

[B23] Doumith M. Ellington M. J. Livermore D. M. Woodford N. (2009). Molecular mechanisms disrupting porin expression in ertapenem-resistant klebsiella and *Enterobacter* spp. clinical isolates from the UK. *J. Antimicrob. Chemother.* 63 659–667. 10.1093/jac/dkp029 19233898

[B24] Falgenhauer L. Imirzalioglu C. Ghosh H. Gwozdzinski K. Schmiedel J. Gentil K. (2016). Circulation of clonal populations of fluoroquinolone-resistant CTX-M-15-producing *Escherichia coli* ST410 in humans and animals in Germany. *Int. J. Antimicrob. Agents* 47 457–465. 10.1016/j.ijantimicag.2016.03.019 27208899

[B25] Forde B. M. Roberts L. W. Phan M. D. Peters K. M. Fleming B. A. Russell C. W. (2019). Population dynamics of an *Escherichia coli* ST131 lineage during recurrent urinary tract infection. *Nat. Commun.* 10:3643. 31409795 10.1038/s41467-019-11571-5 PMC6692316

[B26] Francisco A. P. Vaz C. Monteiro P. T. Melo-Cristino J. Ramirez M. Carrico J. A. (2012). PHYLOViZ: phylogenetic inference and data visualization for sequence based typing methods. *BMC Bioinformatics* 13:87. 10.1186/1471-2105-13-87 22568821 PMC3403920

[B27] Hassuna N. A. Khairalla A. S. Farahat E. M. Hammad A. M. Abdel-Fattah M. (2020). Molecular characterization of extended-spectrum beta lactamase-producing *E. coli* recovered from community-acquired urinary tract infections in Upper Egypt. *Sci. Rep.* 10:2772. 32066805 10.1038/s41598-020-59772-z PMC7026060

[B28] Hojabri Z. Mirmohammadkhani M. Kamali F. Ghassemi K. Taghavipour S. Pajand O. (2017). Molecular epidemiology of *Escherichia coli* sequence type 131 and its H30/H30-Rx subclones recovered from extra-intestinal infections: first report of OXA-48 producing ST131 clone from Iran. *Eur. J. Clin. Microbiol. Infect. Dis.* 36 1859–1866. 10.1007/s10096-017-3021-9 28550370

[B29] Ibrahim R. A. Teshal A. M. Dinku S. F. Abera N. A. Negeri A. A. Desta F. G. (2018). Antimicrobial resistance surveillance in Ethiopia: implementation experiences and lessons learned. *Afr. J. Lab. Med.* 7:770. 30568898 10.4102/ajlm.v7i2.770 PMC6295752

[B30] Johnson J. R. Menard M. Johnston B. Kuskowski M. A. Nichol K. Zhanel G. G. (2009). Epidemic clonal groups of *Escherichia coli* as a cause of antimicrobial-resistant urinary tract infections in Canada, 2002 to 2004. *Antimicrob. Agents Chemother.* 53 2733–2739. 10.1128/aac.00297-09 19398649 PMC2704706

[B31] Johnson J. R. Porter S. Thuras P. Castanheira M. (2017). The pandemic H30 subclone of sequence type 131 (ST131) as the leading cause of multidrug-resistant *Escherichia coli* infections in the United States (2011-2012). *Open Forum Infect. Dis.* 4:ofx089. 28638846 10.1093/ofid/ofx089 PMC5473367

[B32] Kazemnia A. Ahmadi M. Dilmaghani M. (2014). Antibiotic resistance pattern of different *Escherichia coli* phylogenetic groups isolated from human urinary tract infection and avian colibacillosis. *Iran. Biomed. J.* 18 219–224. 25326020 10.6091/ibj.1394.2014 PMC4225061

[B33] Lee J. H. Subhadra B. Son Y. J. Kim D. H. Park H. S. Kim J. M. (2016). Phylogenetic group distributions, virulence factors and antimicrobial resistance properties of uropathogenic *Escherichia coli* strains isolated from patients with urinary tract infections in South Korea. *Lett. Appl. Microbiol.* 62 84–90. 10.1111/lam.12517 26518617

[B34] Liu Y. Feng Y. Wu W. Xie Y. Wang X. Zhang X. (2015). First Report of OXA-181-producing *Escherichia coli* in China and characterization of the isolate using whole-genome sequencing. *Antimicrob. Agents Chemother.* 59 5022–5025. 10.1128/aac.00442-15 26014927 PMC4505247

[B35] Magiorakos A. P. Srinivasan A. Carey R. B. Carmeli Y. Falagas M. E. Giske C. G. (2012). Multidrug-resistant, extensively drug-resistant and pandrug-resistant bacteria: an international expert proposal for interim standard definitions for acquired resistance. *Clin. Microbiol. Infect.* 18 268–281. 10.1111/j.1469-0691.2011.03570.x 21793988

[B36] Manges A. R. Geum H. M. Guo A. Edens T. J. Fibke C. D. Pitout J. D. D. (2019). Global extraintestinal pathogenic *Escherichia coli* (ExPEC) lineages. *Clin. Microbiol. Rev.* 32:e00135. 31189557 10.1128/CMR.00135-18 PMC6589867

[B37] Matsumura Y. Yamamoto M. Nagao M. Hotta G. Matsushima A. Ito Y. (2012). Emergence and spread of B2-ST131-O25b, B2-ST131-O16 and D-ST405 clonal groups among extended-spectrum-beta-lactamase-producing *Escherichia coli* in Japan. *J. Antimicrob. Chemother.* 67 2612–2620. 10.1093/jac/dks278 22843833

[B38] Nicolas-Chanoine M. H. Bertrand X. Madec J. Y. (2014). *Escherichia coli* ST131, an intriguing clonal group. *Clin. Microbiol. Rev.* 27 543–574. 10.1128/cmr.00125-13 24982321 PMC4135899

[B39] Nicolas-Chanoine M. H. Blanco J. Leflon-Guibout V. Demarty R. Alonso M. P. Canica M. M. (2008). Intercontinental emergence of *Escherichia coli* clone O25:H4-ST131 producing CTX-M-15. *J. Antimicrob. Chemother.* 61 273–281. 10.1093/jac/dkm464 18077311

[B40] Paterson D. L. Bonomo R. A. (2005). Extended-spectrum beta-lactamases: a clinical update. *Clin. Microbiol. Rev.* 18 657–686. 16223952 10.1128/CMR.18.4.657-686.2005 PMC1265908

[B41] Pehrsson E. C. Tsukayama P. Patel S. Mejia-Bautista M. Sosa-Soto G. Navarrete K. M. (2016). Interconnected microbiomes and resistomes in low-income human habitats. *Nature* 533 212–216. 10.1038/nature17672 27172044 PMC4869995

[B42] Peirano G. Pitout J. D. D. (2019). Extended-spectrum beta-lactamase-producing *Enterobacteriaceae*: update on molecular epidemiology and treatment options. *Drugs* 79 1529–1541. 10.1007/s40265-019-01180-3 31407238

[B43] Peirano G. Van Der Bij A. K. Freeman J. L. Poirel L. Nordmann P. Costello M. (2014). Characteristics of *Escherichia coli* sequence type 131 isolates that produce extended-spectrum beta-lactamases: global distribution of the H30-Rx sublineage. *Antimicrob. Agents Chemother.* 58 3762–3767. 10.1128/aac.02428-14 24752265 PMC4068522

[B44] Piazza A. Comandatore F. Romeri F. Pagani C. Floriano A. M. Ridolfo A. (2018). First report of an ST410 OXA-181 and CTX-M-15 coproducing *Escherichia coli* clone in Italy: a whole-genome sequence characterization. *Microb. Drug Resist.* 24 1207–1209. 10.1089/mdr.2017.0366 29473791

[B45] Pietsch M. Eller C. Wendt C. Holfelder M. Falgenhauer L. Fruth A. (2017). Molecular characterisation of extended-spectrum beta-lactamase (ESBL)-producing *Escherichia coli* isolates from hospital and ambulatory patients in Germany. *Vet. Microbiol.* 200 130–137. 10.1016/j.vetmic.2015.11.028 26654217

[B46] Pitout J. D. (2008). Multiresistant *Enterobacteriaceae*: new threat of an old problem. *Expert. Rev. Anti. Infect. Ther.* 6 657–669. 10.1586/14787210.6.5.657 18847404

[B47] Quinones D. Aung M. S. Carmona Y. Gonzalez M. K. Pereda N. Hidalgo M. (2020). High prevalence of CTX-M type extended-spectrum beta-lactamase genes and detection of NDM-1 carbapenemase gene in extraintestinal pathogenic *Escherichia coli* in Cuba. *Pathogens* 9:65. 10.3390/pathogens9010065 31963265 PMC7168674

[B48] Ramadan A. A. Abdelaziz N. A. Amin M. A. Aziz R. K. (2019). Novel blaCTX-M variants and genotype-phenotype correlations among clinical isolates of extended spectrum beta lactamase-producing *Escherichia coli*. *Sci. Rep.* 9:4224. 30862858 10.1038/s41598-019-39730-0 PMC6414621

[B49] Reid C. J. Demaere M. Z. Djordjevic S. P. (2019). Australian porcine clonal complex 10 (CC10) *Escherichia coli* belong to multiple sublineages of a highly diverse global CC10 phylogeny. *Microb. Genom.* 5:e000225. 10.1099/mgen.0.000225 PMC6487311 30303480

[B50] Roer L. Overballe-Petersen S. Hansen F. Schonning K. Wang M. Roder B. L. (2018). *Escherichia coli* sequence type 410 is causing new international high-risk clones. *mSphere* 3:e337. 10.1128/mSphere.00337-18 PMC6052333 30021879

[B51] Saitou N. Nei M. (1987). The neighbor-joining method: a new method for reconstructing phylogenetic trees. *Mol. Biol. Evol.* 4 406–425. 3447015 10.1093/oxfordjournals.molbev.a040454

[B52] Schaufler K. Semmler T. Wieler L. H. Wohrmann M. Baddam R. Ahmed N. (2016). Clonal spread and interspecies transmission of clinically relevant ESBL-producing *Escherichia coli* of ST410–another successful pandemic clone? *FEMS Microbiol. Ecol.* 92:fiv155. 10.1093/femsec/fiv155 26656065

[B53] Schwaber M. J. Navon-Venezia S. Schwartz D. Carmeli Y. (2005). High levels of antimicrobial coresistance among extended-spectrum-beta-lactamase-producing *Enterobacteriaceae*. *Antimicrob. Agents Chemother.* 49 2137–2139. 10.1128/aac.49.5.2137-2139.2005 15855548 PMC1087677

[B54] Smet A. Martel A. Persoons D. Dewulf J. Heyndrickx M. Claeys G. (2010). Characterization of extended-spectrum beta-lactamases produced by *Escherichia coli* isolated from hospitalized and nonhospitalized patients: emergence of CTX-M-15-producing strains causing urinary tract infections. *Microb. Drug Resist.* 16 129–134. 10.1089/mdr.2009.0132 20370505

[B55] Teklu D. S. Negeri A. A. Legese M. H. Bedada T. L. Woldemariam H. K. Tullu K. D. (2019). Extended-spectrum beta-lactamase production and multi-drug resistance among *Enterobacteriaceae* isolated in Addis Ababa, Ethiopia. *Antimicrob. Resist. Infect. Control* 8:39. 30815254 10.1186/s13756-019-0488-4 PMC6377715

[B56] Tenaillon O. Skurnik D. Picard B. Denamur E. (2010). The population genetics of commensal *Escherichia coli*. *Nat. Rev. Microbiol.* 8 207–217. 10.1038/nrmicro2298 20157339

[B57] Tong Y. Sun S. Chi Y. (2014). Virulence genotype and phylogenetic groups in relation to Chinese herb resistance among *Escherichia coli* from patients with acute pyelonephritis. *Afr. J. Tradit. Complement Altern. Med.* 11 234–238. 10.4314/ajtcam.v11i3.33 25371588 PMC4202444

[B58] Tooke C. L. Hinchliffe P. Bragginton E. C. Colenso C. K. Hirvonen V. H. A. Takebayashi Y. (2019). Beta-lactamases and beta-lactamase inhibitors in the 21st century. *J. Mol. Biol.* 431 3472–3500. 30959050 10.1016/j.jmb.2019.04.002 PMC6723624

[B59] Tsai Y. K. Liou C. H. Fung C. P. Lin J. C. Siu L. K. (2013). Single or in combination antimicrobial resistance mechanisms of *Klebsiella pneumoniae* contribute to varied susceptibility to different carbapenems. *PLoS One* 8:e79640. 10.1371/journal.pone.0079640 24265784 PMC3827147

[B60] Vandepitte J. Engbaek K. Rohner P. Piot P. Heuck C. C. Rohner P. (2003). *Basic Laboratory Procedures in Clinical Bacteriology*, 2nd Edn. Geneva: World Health Organization.

[B61] Wang S. Zhao S. Y. Xiao S. Z. Gu F. F. Liu Q. Z. Tang J. (2016). Antimicrobial resistance and molecular epidemiology of *Escherichia coli* causing bloodstream infections in three hospitals in Shanghai, China. *PLoS One* 11:e0147740. 10.1371/journal.pone.0147740 26824702 PMC4733056

[B62] Wellington E. M. Boxall A. B. Cross P. Feil E. J. Gaze W. H. Hawkey P. M. (2013). The role of the natural environment in the emergence of antibiotic resistance in gram-negative bacteria. *Lancet Infect. Dis.* 13 155–165. 23347633 10.1016/S1473-3099(12)70317-1

[B63] Williams P. C. M. Isaacs D. Berkley J. A. (2018). Antimicrobial resistance among children in sub-Saharan Africa. *Lancet Infect. Dis.* 18 e33–e44. 29033034 10.1016/S1473-3099(17)30467-X PMC5805911

[B64] Wirth T. Falush D. Lan R. Colles F. Mensa P. Wieler L. H. (2006). Sex and virulence in *Escherichia coli*: an evolutionary perspective. *Mol. Microbiol.* 60 1136–1151. 10.1111/j.1365-2958.2006.05172.x 16689791 PMC1557465

[B65] Woodford N. Turton J. F. Livermore D. M. (2011). Multiresistant gram-negative bacteria: the role of high-risk clones in the dissemination of antibiotic resistance. *FEMS Microbiol. Rev.* 35 736–755. 10.1111/j.1574-6976.2011.00268.x 21303394

[B66] Ye J. Mcginnis S. Madden T. L. (2006). BLAST: improvements for better sequence analysis. *Nucleic Acids Res.* 34 W6–W9. 16845079 10.1093/nar/gkl164 PMC1538791

[B67] Zeynudin A. Pritsch M. Schubert S. Messerer M. Liegl G. Hoelscher M. (2018). Prevalence and antibiotic susceptibility pattern of CTX-M type extended-spectrum beta-lactamases among clinical isolates of gram-negative bacilli in Jimma, Ethiopia. *BMC Infect. Dis.* 18:524. 10.1186/s12879-018-3436-7 30342476 PMC6196031

[B68] Zhou Z. Alikhan N. F. Mohamed K. Fan Y. Agama Study G. Achtman M. (2020). The enterobase user’s guide, with case studies on *Salmonella* transmissions, yersinia pestis phylogeny, and *Escherichia* core genomic diversity. *Genome Res.* 30 138–152. 10.1101/gr.251678.119 31809257 PMC6961584

